# Nursing

**Published:** 1873-07

**Authors:** 


### NURSING.

BY B. B.

It is noticeable how few persons are good nurses—possessing tender and sympathetic natures which bring them into immediate help and worth in a sick room.

In these modern times, when doctors are relying more upon careful attendance upon the sick man's feelings than upon the guess work of doses, this is a very important matter. Both cure, but the skillful nurse excels the patent pills and globules.

In order then to render every assistance to those who would be glad to know what to do if called upon—as every one is liable to be—to watch over and care for some friend, I offer the following suggestions.

First, I will remark of the provision for the sick in every household. Attention should be given in every family to the possible wants of its members in sickness, A sick room or nursery should be provided in which especial care has been taken to have nothing which may irritate the senses of the sick. In health we do not regard a hundred little things which are an annoyance when we are ill.

A conspicuous or peculiar pattern of paper upon the walls should be particularly avoided, for strange figures or shapes are conjured up by a morbid sight and imagination, from suggestive papering.

A creaking or rattling window blind, from the very monotony of its sounds and the inability of the sick man to turn from it, will produce a high state of nervousness.—So also will a rustling window shade which slips in and out through a partially opened sash.

Familiar noises of the active household attending to the necessary family duties should be guarded against if possible; the smell of kitchen should *never* enter a sick room.

Do not call those mere trifles—they are not. Do you care to "put yourself in his place" and test their importance? I could wish you no worse misery than to be helplessly laid on a sick bed and obliged to endure some of these "trifles," as you may call them. I have known of strong minded men who have cried like babies because some one has forgotten to close the door tightly, and its chucking together in a current of air had soon become so wearisome that all self-control was gone.

Secondly, I wish to suggest as to conduct in the sick room. The dress of the nurse should be so simple and plain, as not to attract attention except for its entire neatness and becoming appearance. A plain material which will not rustle when moving about the room is the best.

Do not enter the room with an air suggestive of your strength and health, inducing a comparison with his weakness in the mind of the patient.

Be in harmony with the condition of the sick, thereby creating a feeling of support for his feebleness.

Do not sit in a rocking chair—in fact I would not have one in the room unless the patient could use it—and commence to swing yourself back and forth in it; it may annoy more than you think. Of course no one would think of wearing heavy boots or shoes in such a place. Slippers are heavy enough for the little walking which is necessary.

Learn to think for, as well as of, your charge. Be quick to notice whether what you do, or do not do, annoys in any manner. Do not expect to be told, but understand without any hints. Recall to mind your own experience and avoid causing any discomfort.

Never contradict or enter into an argument as to the right or wrong of any whim which you may encounter. You must possess a rare tact to out-talk without irritating, for remember that reason seldom enters into the vagaries of the sick. They want because they want, just as a child does. It is almost impossible to convince a person who in health possesses a strong mind and is used to self-judgment, that his various desires are whimsical and therefore it is of but little consequence whether they are filled or not. Some diseases seem to, and in fact do, set the mind in a greater state of activity than when in health.

The appetite is often very fickle, requiring great care and art to please it. In such cases the more simple, and therefore the more apt to be forgotten foods, will usually be most acceptable. Fancy things are likely to be stale and insipid. Do not previously inform your patient that you will have some certain thing prepared for him in such a time. Make a pleasant surprise by offering a tempting dish of something which has not been anticipated for hours.

Finally, never lose your patience and exhibit a feeling of irritation at any treatment or lack of appreciation of your services. When health returns you will experience a pleasure in thinking that you have contributed in restoring it.

Pay attention to the instruction given with the medicines and be sure that no mistakes occur at your hands. Familiarize yourself with the nature of the medicines which you give, and endeavor to ascertain whether they are effective. In this way you can assist the doctor, who, necessarily spending but a short time at the bedside of his patient, must depend upon you for information as to the condition of your mutual charge.

All of these suggestions are of a very simple kind, yet any one of experience I believe will assent to their importance. If you who read them did not know them before, then I hope that they will remain with you and be of service if you are called to minister to the sick.

